# Incised Wound of the Left Side, between the Eighth and Ninth Ribs, Followed by Protrusion of the Stomach and Strangulation of the Organ; Reduction and Recovery of the Patient

**Published:** 1853-06

**Authors:** W. W. Hart

**Affiliations:** Carrollton, Miss.


ART. IV.—*Incised Wound of the Left Side, between the eighth and ninth ribs, followed by protrusion of the Stomach and strangulation of the organ;* Reduction and recovery of the patient. By W. W. HART, M. D., of Carrollton, Miss.

In April, twelve months ago, I was summoned in great haste, by Dr. A. Ewing, (who had been previously called in,) to a negro man aged about twenty-five years, the property of Mr. J. E. Harnon, residing five miles east of this place, who, in a rencounter with his overseer, received an incised wound in his left side, about three inches in length, between the eighth and ninth ribs. The wound was inflicted in the beginning of the affray, with a pocket knife, the blade of which was about four inches in length. The negro, notwithstanding, continued to resist, and by the violent exertions made caused the stomach to protrude. He finally became exhausted and was carried to the house. On my arrival I found the stomach protruding out of the wound, forming a tumor about the size of the two fists placed with the thumbs and fingers opposite; considerably strangulated, and perfectly tense with air and undigested food. I made several efforts to reduce it by pressure, but was unable to accomplish it in consequence of the narrow space between the ribs, compared with the size of the tumor. I then handed Dr. E. a probe-pointed bistoury and requested him to make the incision longer, hoping by this means to effect a reduction. He increased its length about one inch, but nothing was accomplished by the procedure, save the escape of a little air, upon pressure, which was immediately ejected through the œsophagus.

Our efforts thus far having been without effect, and knowing that something must speedily be done, we concluded to make an incision into the stomach and empty it of its contents. Accordingly an incision about two inches in length was made into the organ and the contents removed.

We then closed the wound by the interrupted suture and replaced the organ. This being done, it was ascertained that the lower margin of the left lung was slightly wounded, as was evinced by the escape of air at each inspiration. The diaphragm and omentum were also wounded.

We removed all the coagulated blood within reach, and closed the external wound by means of a few stitches and adhesive strips. The patient was then placed in bed and kept in a state of perfect rest, on low diet, and the bowels opened by enemata. On the second day he was resting quietly; passed a pleasant night, and continued to improve without any unfavorable symptom whatever.

In four weeks he was perfectly well and pursuing his ordinary labors.

April 19th, 1853.

